# Data Improvement Model Based on ECG Biometric for User Authentication and Identification

**DOI:** 10.3390/s20102920

**Published:** 2020-05-21

**Authors:** Alex Barros, Paulo Resque, João Almeida, Renato Mota, Helder Oliveira, Denis Rosário, Eduardo Cerqueira

**Affiliations:** Computer Science Faculty, Federal University of Pará, Belém 66075-110, Brazil; paulo.resque.souza@icen.ufpa.br (P.R.); joao.freitas.almeida@icen.ufpa.br (J.A.); renato.mota@icen.ufpa.br (R.M.); heldermay@ufpa.br (H.O.); denis@ufpa.br (D.R.); cerqueira@ufpa.br (E.C.)

**Keywords:** authentication, security, biometric, ECG, random forest, wearables

## Abstract

The rapid spread of wearable technologies has motivated the collection of a variety of signals, such as pulse rate, electrocardiogram (ECG), electroencephalogram (EEG), and others. As those devices are used to do so many tasks and store a significant amount of personal data, the concern of how our data can be exposed starts to gain attention as the wearable devices can become an attack vector or a security breach. In this context, biometric also has expanded its use to meet new security requirements of authentication demanded by online applications, and it has been used in identification systems by a large number of people. Existing works on ECG for user authentication do not consider a population size close to a real application. Finding real data that has a big number of people ECG’s data is a challenge. This work investigates a set of steps that can improve the results when working with a higher number of target classes in a biometric identification scenario. These steps, such as increasing the number of examples, removing outliers, and including a few additional features, are proven to increase the performance in a large data set. We propose a data improvement model for ECG biometric identification (user identification based on electrocardiogram—DETECT), which improves the performance of the biometric system considering a greater number of subjects, which is closer to a security system in the real world. The DETECT model increases precision from 78% to 92% within 1500 subjects, and from 90% to 95% within 100 subjects. Moreover, good False Rejection Rate (i.e., 0.064003) and False Acceptance Rate (i.e., 0.000033) were demonstrated. We designed our proposed method over PhysioNet Computing in Cardiology 2018 database.

## 1. Introduction

In the last few years, wearable technology has reached beyond the realms of science fiction since computing devices stopped being an item used only in our homes and workplaces, or carried in our bags and pockets. Presently, we can wear those devices and use them connected to the Internet, providing a variety of applications [1]. Connectivity becomes easy and increasingly crucial for many of us, and also access to data and knowledge has been made affordable, manageable, and convenient. On the other hand, the concern of how our data can be exposed gains a lot of attention, as these devices are used to do many tasks and store a significant amount of personal data. In this context, wearable devices enable the collection of biometrics and/or environmental interaction data, such as pulse rate, electrocardiogram (ECG), electroencephalogram (EEG), photoplethysmogram (PPG), O2 saturation, and other for remote controllers or assessments for sports players, exercise metrics, weather information, and others [2,3]. In this way, wearable devices provide a wide range of new applications and can be used as one sensor to collect a new biometric sign.

Current authentication systems started to be based on something you are (biometric), and not something you have (cards or keys) or something you know (password), since cards, keys or passwords can be lost, stolen, discovered, or copied [4]. This is because biometric signs collected by wearable devices are focused on intrinsic characteristics of the person, requiring their physical presence, and minimizing the probability of success of possible impostors. Biometrics can be used either to identify or authenticate a subject based on a measurement of one or several biometric traits [5]. Despite it having some advantages compared to passwords and traditional security measures, fingerprint, voice, and face were also proved to be vulnerable years ago [6]. Considering this, authentication systems based on physiological features such as ECG, PPG, and EEG have the advantage of assuring user’s mobility with a higher certainty of an individual’s liveness [7], where ECG is very useful for user identification [7]. Individually, ECG excels with other physiological traits in some respects and provides universality, uniqueness, hidden nature, permanent and simple acquisition [8]. Hence, ECG collected by wearable devices has been used by many researchers for biometric identification, since it has features that are unique to an individual, such as statistical, morphological, and wavelet [9].

Different Machine Learning (ML) algorithms performed almost well for complex problems, such as user identification based on ECG data, once they receive enough data [10]. In this sense, to validate the ECG as biometric for user authentication, ML models should be trained with a higher number of subjects, simulating the scenario presented in real-world applications. Most of the existing works on ECG for user authentication and identification [7,8,11,12,13] analyzed datasets with 20 to 290 people records. Such amount of data is not enough to provide user authentication and identification system with scalability, such as the system operation today. Considering, for example, an ordinary building entrance control system, where a business building easily has thousands of users every day. Some companies have buildings in different cities and/or countries, achieving again, thousands of possible users aiming to get authenticated into the intern system. Considering a web application related to transportation mobility, the number of users can achieve millions in a metropolis. Due to these facts, an evaluation of the ECG signal working with a great number of people is really necessary. We selected a dataset with almost 2 thousand people which is close to real applications in terms of the number of users. This dataset allows us to evaluate how a system would work with more data. Based on our preliminary evaluation for user identification based on ECG, the performance reduces as soon as the number of target classes increases. For instance, precision starts around 78% with 100 subjects and ends close to 60% using 1500. This is because the ML model needs more features to generalize a great number of classes. However, more features would become the fitting process more complex and slower. In this context, data improvements (i.e., increasing the number of examples, remove outliers, data augmentation, and include a few additional features) must be applied in order to achieve good results with a greater number of subjects. Therefore, the developer should spend more time improving the data set than training ML models, since data matters more than the algorithm [10].

Find enough labeled samples of data has become one of the bigger challenges for researchers. Not having enough quality labeled data will generate overfitting, which means that the system is highly biased to the data it has seen in the training set and, therefore will not be able to generalize the learned model to any other samples [14]. For image recognition applications, mirroring, scaling, cropping, rotating, and so on. are legitimate augmentation techniques as minor changes due to these techniques do not alter the label of the image as they may happen in real-world observation [15]. However, these label-preserving transformations can affect and actually shadow the features when working with time-series medical data. Novel approaches must be proposed to improve the size of the dataset for biometric systems using biosignals such as ECG.

In this article, we propose a data improvement model to improve the data set quality, resulting in performance improvements during the ML process for user identification based on ECG signals, called of DETECT. Each step of DETECT contribute to the increasing metrics results, we believe they must be used together to increase the quality of data and avoid bias. Many algorithms can achieve really good results but they do not have any set of basics transformation to be applied to raw data to guarantee a minimum quality. The filtering steps are good to remove noise, but the outliers still can affect the results. In the proposed model, we increased number of examples using a particular Data Augmentation step, introduced an outlier removal using Outlier to discard the data with values out of IQR, and also considered a different set of features. To validate DETECT, we choose the PhysioNet Computing in Cardiology 2018, due to its great number of subjects collected, i.e., almost 2 thousand. We also investigate one of the most used ML algorithms for user identification, i.e., Random Forest (RF). Based on that, we analyze the accuracy of DETECT to classify people in continuous authentication and identification scenario. Evaluation results show the potential of the DETECT, which increased precision in 20% for 100 subjects, and 16% considering 1500 subjects compared to the same classification process without any data improvement. Our proposed model has a set of additional modules delivering some improvement to the system at the end of each step, such as outlier removal, data augmentation, and increased number of features. In this way, the main contributions of this article can be summarized as follows: (i) data improvement model to improve the data set quality for user authentication and identification based on ECG signals. (ii) evaluation considering PhysioNet Computing in Cardiology 2018 database, due to its great number of subjects collected. (iii) Evaluate the performance of DETECT using generic ML metrics, such as Precision, Recall, and F1 score. We also used biometric identification metrics, namely, False Acceptance Rate (FAR) and False Rejection Rate (FRR).

The remainder of this article is structured as follows. Section 2 outlines an overview of different techniques with ECG for user identification. Section 3 outlines some ECG data sets and the DETECT model. Section 4 introduces the achieved results. Section 5 presents the concluding remarks and future works.

## 2. Related Work

Zhang and Wu [16] considered an authentication using ECG collected from two fingers electrodes in association with a smartphone application. They selected 85 subject’s ECG records from widely used public databases on PHYSIONET [17]. For identification, the system fulfills a classification using Support Vector Machine (SVM) and Neural Networks (NN). A voting mechanism required more than half of the voters to validate the testing person. This work achieved 97.55% of accuracy and performed authentication in 4 s.

Zhang, Y et al. [8] combined fiducial and non-fiducial features (hybrid approach) to enhance accuracy for authenticating many users. This work considered the PQRST peaks as the main fiducial features, i.e., segments PQ, QR, RS, and ST duration, PQ, PT, and SQ amplitudes determined by wavelet transform. For non-fiducial-based features, they defined the ECG signal as a matrix (X), obtained the Gramian matrix multiplying $X^{T}X$, and finally obtained the features from the eigenvalues and eigenvectors from the Gramian matrix. They improve efficiency by increasing the number of features, leading to increased computational effort [9].

Camara et al. [13] focused on Continuous Authentication (CA) using biosignals, where the user is authenticated every period of time, ensuring the continued presence of the user. The samples are classified using the Decision Tree (DT), SVM, and other ML algorithms. They achieved accuracy ranging from 97.4% to 97.9%, depending on some parameters by using recordings from 10 individuals from the MIT-BIH Normal Sinus Rhythm DataBase (NSRDB) [17].

Zhang, Q et al. [11] introduced a novel wavelet domain multi-resolution convolutional neural network approach (MCNN) for ECG biometric identification, which considers the total of 220 people. The achieved an identification rate of 96.5% for typical data sets, 90.5% for abnormal, and 93.5% for all data sets. They used a technique of getting random windows from ECG segments to increase data representation to cope with a data set with a small number of samples.

Labati et al. [18] proposed a similar use of CNN as [11]. The contribution was to introduce a method to binarize ECG templates that permits a reduction of the matching time and apply template protection techniques. Their approach extracts a set of *m* QRS complexes from ECG samples of short duration, and joins them in the signal V. In closed-set identification, a CNN processes V and indicates who is the closest registered user. In identity verification and periodic re-authentication, the CNN processes V to obtain a biometric template T. A simple distance measures, such as Euclidean or cosine distances is used to compute the matching score. CNN’s architecture still seems to be very complex to be processed in wearable devices with computer power constraints. Most of studies that considered the application of deep learning strategies for ECG analysis were focused on the classification of heartbeats in healthy and non-healthy using techniques such as CNNs, autoencoders, or deep belief networks.

Zhang, et al. [19] used a pre-trained NN to perform a specific task (e.g., classification) on a particular data set (e.g., a set of images). They use the beneficial characteristics of the Google Inception Net and residual neural network (ResNet) to their proposed architecture. Also, no reference point detection or time-consuming handcrafted feature engineering efforts are required. Four public data sets were tested, including Physikalisch-Technische Bundesanstalt (PTB), Combined measurement of ECG, Breathing, and Seismocardiograms DataBase (CEBSDB), NSRDB, and MIT-BIH Arrhythmia DataBase (MITDB). Their results were for the nearest neighbor classifier (NNC) achieved an accuracy of 97.7%, which SVM achieved 98.7% on the three data sets.

Cao et al. [20] highlighted the success of the machine learning model is related to the availability of a rich dataset. They proposed a novel data augmentation strategy specifically designed for analysis of ECG. It was based on duplication, concatenation, and resampling of ECG episodes. The strategy can increase the diversity of samples as well as balance the number of samples in each category, which facilitates the deep learning models in extracting the features from the dataset. They examined the results using the public dataset from the CinC challenge 2017. This dataset focused on the classification of the ECG signals into four types of normal and abnormal classes. It contained 8528 single-lead ECG recordings with different lengths (9 s–61 s). All the recordings were collected using the AliveCor device (AliveCor Inc., Mountain View, CA, USA), were sampled at 300 Hz. Although this work is not related to biometric authentication, it demonstrated that augmentation techniques should be applied to improve small datasets. We explored data augmentation in our proposed model.

Alotaiby et al. [21] uses simple statistics for feature extraction, including the mean, standard deviation, median, maximum value, minimum value, range, interquartile range, interquartile first quarter (Q1), interquartile third quarter (Q3), kurtosis, and skewness of the ECG signal. The dataset used was the PTB ECG database again. The authors achieved an average accuracy of 99.61% using their band-based approach from single limb lead using Random Forest Classifier. They used a data segment length of 7 s, which guarantee a good number of heartbeat but takes too long to be acquired and can bother the user.

Pouryayevali et al. [22] proposed a set of standards for ECG signal recording and presented the UofT ECG Database (UofTDB) to evaluate the performance of various ECG biometric methods. They recorded ECG signals from 1020 subjects captured from fingertips similar to Lead I configuration. They also recorded under different postures (sit, stand, supine, and tripod) and exercise conditions. The Vernier ECG sensors were used with a sampling rate of 200 Hz. They tried to demonstrate that there are factors that affect biometric accuracy over time. Their database has a good number of people, unfortunately, it is a private database.

Based on our analysis of the state-of-the-art, we conclude that most of the existing works on user identification/authentication based on ECG consider databases from PHYSIONET, as most of them were collected from medical studies. However, databases from PHYSIONET do not have large number of people when compared with large government’s biometric database already running in some countries, such as India [23], Brazil [24], and China. For instance, India has a database containing fingerprint and eye scans of more than 1.2 billion of people. This kind of database is usually used in election system to standardize official documents, to provide security in school exams, and in healthcare systems. In this way, we looked for bigger dataset to investigate how a user identification/authentication based on ECG would work with greater number of target classes such as UOFTDB dataset, which is not publicly available. It was observed that tuning in the algorithm parameters already gives good results with few target classes, but, in practice, working with bigger dataset, it would not be enough. A data improvement, such as increasing the number of examples, remove outliers, and include additional features, will be required to achieve good results. Actually, sometimes the data collected will not have the minimum quality or number of samples and the user will be requested to collect it again. The filtering step is the only consensus when we talk about data improvement [8,9,11,16], as noise is expected to be included during acquire process. A few data improvements were used, such as Z-score normalization in Zhang et al. [8] over PTB, ECGDB, NSRDB, and CEBSDB datasets. They also applied a special segmentation technique, but outliers were not removed, probably affecting the results. In Zhang et al. [11], a blind split was applied into signal segments over eight diverse datasets. Then all the recordings were scaled to be between 0 and 1 and subtracted by their mean to balance their contribution in the algorithm training phase. We noticed a lack of mature techniques to improve the quality of data. Fingerprint-based and face-based identification are more mature in these aspects. More efforts still need to be put in this aspect while more and more ECG datasets are becoming available, even the amount of data is still not comparable to the fingerprint data. Hence, data improvement is necessary to increase and to provide a baseline to use ECG as a trustable biometric system. Table 1 summarizes the analyzed works related to ECG Biometric systems.

## 3. DETECT Model

In this section, we describe the DETECT model for ECG biometric authentication and identification system. In this way, we introduce a set of steps to improve the ECG data set quality, resulting in performance improvements during the authentication and identification process.

### 3.1. Evaluation Scenario

**Figure 1:**
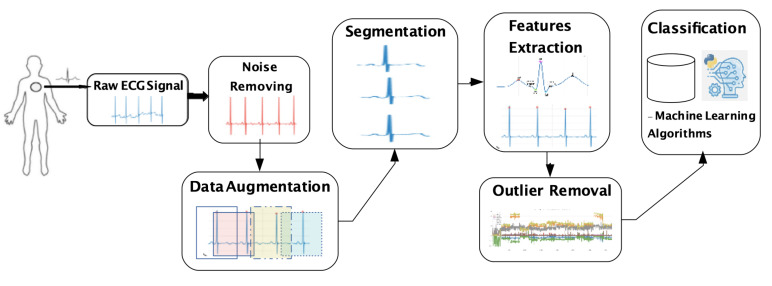
Overview of all processing steps of DETECT.

ECG has been used by many researchers in the biometric identification system, since it has features that are unique to each individual, such as statistical, morphological, and wavelet features [7]. Hence, ECG for user identification is divided into five steps: Raw ECG signal acquisition, noise removing, segmentation (and possibly data augmentation), features extraction (and possibly outlier removal), and classification, as depicted in Figure 1.

A user authentication system checks a set of features against a profile that already exists in the data set linked to that individual’s credentials, which is known as one to one matching system. For identification, the system checks collected features against all other templates in the database, which is described as a one to many matching systems. Hence, the authentication process answers if the subject is who he or she claims to be, while identification answers if he or she is somebody registered in a previous database.

As wearable devices can capture the signal continuously, it could be used to continuous authentication in the future and we believe that they will be the main source of sensing data soon. Generally, the ECG signal might be captured mainly through wearable devices, which must be pre-processed to remove noise. ECG signal must be divided into segments; it was applied split up blindly into signal segments with an equal length of 3 s without leveraging any heartbeat location information. The idea is to design a solution that does not spend computational resources finding the QRS complex to segment the data. The QRS complex will be found only in the feature extraction step. In this context, data augmentation is an alternative approach to increase the number of possible segments. After segmentation, we apply the feature extraction, and the resulting features are processed to form a template to compare with the authorized user template. Specifically, it extracts the user’s characteristics from the ECG for the authentication process, but outliers caused by noise or misplacement in the acquisition step can compromise the classification. In this sense, an outlier removal step is needed. Finally, classification is applied to distinguish genuine and imposer vital signal data. In this sense, feature extraction is the most important step, because it is when the user’s characteristics are extracted from the vital signal for the authentication process.

### 3.2. ECG Database

ECG records electrical activity generated by the heart in a non-invasive way, where the heart beats generate polarization and depolarization waves in the muscle fibers, and thus ECG registers the heart activity based on the resulted potential differences. A typical ECG signal has six remarkable points of a single healthy heartbeat: P, Q, R, S, T, and U, which can be used for user identification [25]. The stimulus produced during depolarization and re-polarization generates such behavior [26]. The depolarization phases correspond to the P-wave (atrial depolarization) and QRS-wave (ventricles depolarization). The re-polarization phases correspond to the T wave and U wave (ventricular re-polarization) [9].

In this context, sometimes the biggest challenge is not to develop a model or choose ML classifier; the main problem is to find a data set closer to real-world situations. There are many data sets available and they differ in the number of subjects, healthy or not, and type of acquisition sensor used, inside or outside the hospital environment, and so on. By analyzing these ECG databases, we choose the PhysioNet Computing in Cardiology 2018 database [17], called *“You Snooze You Win”*. This is because it captures a variety of physiological signals recorded during the user slept through the night, including EEG, electrooculography (EOG), electromyography (EMG), ECG, and oxygen saturation (SaO2).

Data were collected by the Massachusetts General Hospital’s (MGH) Computational Clinical Neurophysiology Laboratory (CCNL), and the Clinical Data Animation Laboratory (CDAC). Moreover, it is the biggest publicly available ECG data set, including data from almost 2 thousand subjects. Specifically, data set includes 1985 subjects monitored at an MGH sleep laboratory for the diagnosis of sleep disorders. In this way, PhysioNet data set provides the opportunity to evaluate how the biometric system based on ECG performs when the number of people increases. To the best of our knowledge, this is the first time the PhysioNet Computing in Cardiology 2018 database is used in authentication or identification research.

Most available datasets have some constraints that limit its application in practical simulations. We should mention the persistence of an individual’s ECG characteristics over time. In addition, the shape of ECG waveforms is determined primarily by human anatomical features, where the natural variability of which is measured in years. Over a short time, ECG cycles have small qualitative or quantitative variations more likely caused by variations in acquisition procedure and filter distortions. Variations within an hour are almost identical with variations within six months [27]. Of course, there are many other natural and artificial, intentional, and unintentional causes of ECG variability. Some of them are age-related and others related to certain medications that may temporarily change the configuration of the cardiac cycle.

Given this, it is likely that periodic updates of the training set records and classifier retraining will be needed as components of an identification system. All these datasets are very useful to evaluate an initial operation of the biometric system, but it will have to be rebuilt several times during its lifecycle. An ECG identification system will face a variety of challenges that are similar to those posed by various attacks on other types of biometric systems [27].

### 3.3. Noise Removing

The original ECG signals are vulnerable to different sources of noise, such as electromagnetic interference, and sudden user movements in regions close to the sensor [28]. Similar to other works [19,22], the raw ECG signals were filtered using a fourth-order bandpass Butterworth filter with cut-off frequencies 1 Hz and 40 Hz. Under 0.5 Hz the signal is corrupted by baseline wander, and over 40 Hz there is distortion due to muscle movement and power-line noise [22].

**Figure 2:**
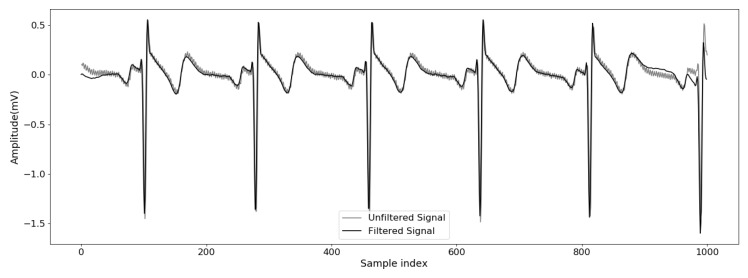
Unfiltered and filtered signal.

As this data was collected in a controlled environment, it is expected that the signal has less noise than ECG collected while the person was driving, doing some movements, and so on. A more sophisticated filtering process should be used when data were acquired from a mobile source. Figure 2 shows both the raw signal with noise and the result of applying the 4th order Butterworth bandpass. In this case, once you know how the waveform of ECG should be, it is easy to see that the waveform was distorted. The choice of filter depends on each signal, and other configurations can perform better considering the noise in the acquire process.

### 3.4. Segmentation and Data Augmentation

ECG is a continuous signal captured from a wearable device in contact with the body. It is usually segmented based on the peaks of the signal to be used as input into the biometric system. In this sense, a blind split up into signal segments was applied with an equal length of 3 s without leveraging any heartbeat location information, similar to that used in Zhang et al. [11]. Considering the practical aspects of registering process, we decided to use only 1 min of each person from the PhysioNet Computing in Cardiology 2018 database. This database has 200 Hz frequency, and thus each segment of 3 s would have at least 600 samples. This window size would be possible to get at least one heartbeat, allowing extraction of more features with less effort since typical heart rate ranges from 40 to 208 beats per minute. Considering the traditional segmentation process, we would get only 20 segments from 60 s. Hence, we need to add a data augmentation step to improve this data.

Data augmentation is the process of increasing the amount and diversity of data. This process helps to increase the amounts of data because sometimes it is not feasible to collect thousands or millions of samples, thus data augmentation increases the size of information and introduces variability. For each recording, we collected an example of a randomly chosen ECG window, which can include a different set of heartbeats and present some signal morphologies depending on each signal window. For instance, we would get more than 20 segments with 3 s each for a 60 s data from a data set. Hence, data augmentation enables random selection of quite more segments. Specifically, we got 80 segments for each subject. This helps to increase the performance of the model by generalizing better and thereby reducing overfitting.

### 3.5. Feature Extraction and Outlier Removal

**Figure 3:**
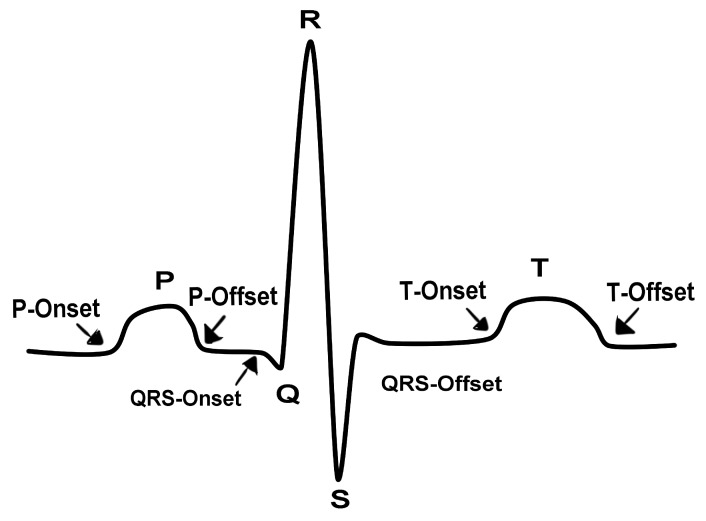
Example of P-QRS-T cycle, with the peaks of all the relevant waveforms.

It is important to extract characteristics from the ECG to use as a template during the authentication process, as shown in Table 2. An ECG signal has many characteristics that could be used, where the smallest number should be chosen to avoid complexity or too much computing [9]. In this context, ECG contains three predominant characteristics used for user authentication: P wave, QRS complex, and T wave, as shown in Figure 3.

The QRS detection algorithm must find all the local maximum points every 3 s, and thus we compare the amplitude values to store the peaks. Initially, the algorithm finds the maximum R peak, and using a threshold of 66% of the maximum value eliminates the points under this value [7]. The remaining points are classified as R Peak. The first local maximum values back and ahead to the R peak are classified as points Q and S, respectively [12]. In this way, the algorithm finds the Q, R, and S amplitude for each beat. Considering that more than one peak is presented in 3 s, we compute the mean and standard deviation of Q, R, and S peaks amplitude, which are our first 6 features. The QRS peak amplitude is calculated by doing the peak R minus peak S amplitude. R wave duration is calculated subtracting the time of S peak minus Q peak. The R-R interval is calculated when more than one R peak is inside the 3 s window.

Besides the QRS complex, we also consider the QRS Onset and Offset, as well as peak P and T points. Onset point of each ECG’s wave is considered the point when the wave starts the slope and offset is the end of a wave. Specifically, the QRS Onset and Offset are computed after Q and S points. Using Q as a reference, the extractor must go back into the raw signal and calculate the greater slope between the new point and the Q point, by using a time window of 40 ms. A similar procedure is done to find the offset, but considering the S point as a reference, and the extractor walks to the end of the array. Finally, we consider a window starting from QRS to find P and T. The most relevant peaks found in this search window is declared P and T on each side of the QRS complex. After collecting these features, it is possible to compute the mean amplitude of the following features: P Onset and Offset, T Onset and Offset, QS distance, QT distance, QRS offset, and QRS onset, and time-related features from these relevant points.

These time-related features are the means of the following: QT interval, ST interval, T wave, PQ segment, ST segment, TP segment and PP interval. These features are simply measuring the time difference between the main waves of the ECG signal or the duration of a single wave. All the discussed features are displayed in Table 2.

Our feature extraction method obtains additional features from the ECG signal. This is because we aim to provide additional information to the prediction model to increase the distinguishing factor between different classes (people). We also included an outlier removal step, which aims to clean up statistically dispersed data features to ensure that the model is not fed with abnormal data. Errors during signal acquisition are the main cause of abnormal data, and this can happen due to factors such as the excessive movement of sensors, electrostatic interference during measurement, removal of sensors improperly during measurement, among others. The removal technique was the InterQuartile Range (IQR). The upper and lower limits of the quartiles were 0.5% for both limits. Any data above or below these thresholds have been discarded. The process was applied to each feature of the data set. This approach is similar to what most of the biometric systems do today, and they require good samples to create a model, if the samples do not have the minimum qualifications required, they are discarded and is necessary to collect data again.

### 3.6. Classification

We consider RF for the classification step since it presents good results without the need for a longer time to fit [12]. RF is a collection of DT trained with randomly selected data, guaranteeing that each tree is slightly different from each other. Each tree may return a distinct result for a given data set. The RF classifies the data based on a voting system involving the results from the individual trees, i.e., count how many trees classified a given feature under a particular class. RF presents a good performance in two critical aspects: anomaly detection and overfitting. This is because during the training process, the outliers will be present in some of the trees but not in all of them, and thus the voting system guarantees the anomalies will be minimized. The voting system also minimizes the effect of overfitting concerning the individual DT.

## 4. Evaluation

In this section, we describe methodology, metrics, and results used to evaluate the DETECT model for user identification based on ECG signal.

### 4.1. Methodology

We evaluated specifically the identification scenario, where a single model is trained to differentiate many users given their ECG signal extracted features as an input. This single model was implemented as a one-vs-rest classifier. The one-vs-rest classifier we used was built on top of instances of random forest classifiers as its base estimators. These base estimators are responsible for learning the features of each person’s ECG signal. Using a one-vs-rest classifier involves splitting the initial 1985 class identification problem into 1985 individual binary classification problems. Then, when the one-vs-rest classifier model is asked to predict a certain class, each of the 1985 base estimators calculates probabilities of this certain class belonging to their own respective class. Finally, the model that outputted the greatest probability is used to indeed predict the class.

Implementation was mostly done using Python’s scikit-learn package and for each scenario tests consisted of the same model: A one-vs-all classifier constituted of random forest classifiers as its base estimators. For each scenario, the dataset was split into training and testing sets in the proportion of 80% for training and the remaining 20% for testing using as randomness control a predefined random state value. Following this, a grid search was performed for tuning the parameters of the random forest classifier base estimator in a 3 subsets cross-validation scheme. All tests were run either on the laboratory server (Intel(R) Xeon(R) Silver 4112 CPU at 2.60 GHz and 64 Gb of RAM) or in Google Colab platform.

**Figure 4:**
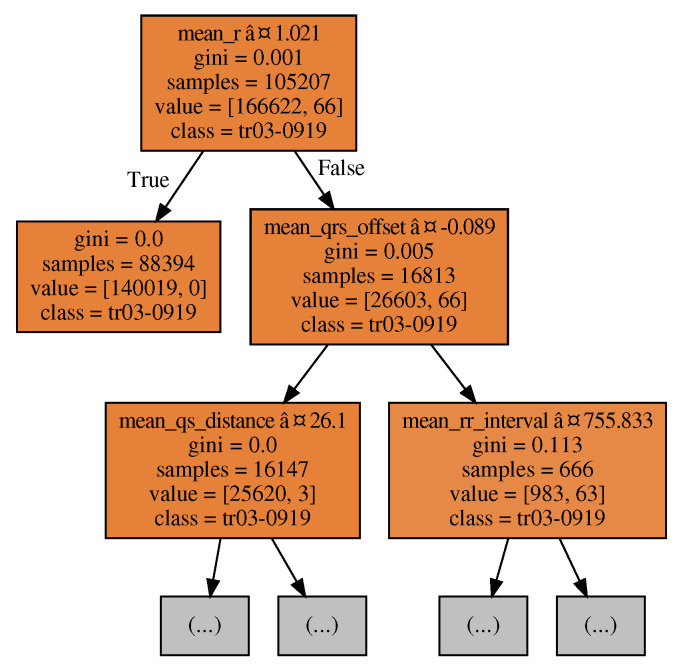
Part of a decision tree from one of the one-vs-all model random forest base estimators.

The parameters and values evaluated were max_depth (60, 80, 100), min_samples_leaf (3, 4, 5), min_samples_split (8, 10, 12) and n_estimators (80, 100, 120). Specifically, the maximum depth of the tree means nodes are expanded until all leaves are pure. The min_samples_leaf is the minimum number of samples required to be at a leaf node. A split point at any depth will only be considered if it leaves at least min_samples_leaf training samples in each of the left and right branches. The min_samples_split is the minimum number of samples required to split an internal node. n_estimators refer to the number of trees in the forest. Setting a random state value was also necessary to bootstrap training samples used when building trees and when sampling features to consider when looking for the best split at nodes of the decision trees). Based on the grid search, we found best parameters for the RF, namely max_depth = 100, min_samples_leaf = 3, min_samples_split = 10, and n_estimators = 120. Figure 4 shows an example of how a RF model with single decision tree might interpret incoming data during the identification task. We also concluded that greater values than the tested here for these parameters indicates classifiers more specialized in predicting the classes. It would be a cost of a considerable increase in memory consumption and a potential overfit of the model. We did not go any further into this relationship (forests robustness) and models still with the aforementioned values.

We analyzed four user identification models based on ECG, namely: Scenario 1—Data without any improvement; Scenario 2—Data with an increased number of examples using Data Augmentation step to increase the number of samples; Scenario 3—Data with an increased number of examples and outlier removal using Outlier Removal step to discard the data with values out of IQR; DETECT model—using the steps of our data improvement model introduced in Section 3.

We select the metrics according to results presented in a confusion matrix to evaluate DETECT model. The confusion matrix tabulates the predicted results against the observations. Specifically, True Positive (TP) standing for correctly accepted instances. False Positive (FP) standing for incorrectly accepted instances. True Negative (TN) standing for correctly rejected instances, and False Negative (FN) standing for incorrectly rejected instances. Based on the confusion matrix, we derive Key Performance Indicators (KPI) to evaluate the model in terms of biometric identification and generic purpose metrics.

We used metrics from the field of biometric identification: False Acceptance Rate (FAR) and False Rejection Rate (FRR) to evaluate the proportion of wrong identifications. FAR evaluates instances that were accepted disguised as classes other than the intended one, while FRR evaluates instances that were incorrectly rejected as a member of a certain class. FAR and FRR are also known in the machine learning evaluation metrics field as false positive rate (FPR) and false negative rate (FNR), respectively (as demonstrated in Equations (1) and (2).

(1) $$FAR=\frac{FP}{FP+TN}$$

(2) $$FRR=\frac{FN}{TP+FN}$$

Also, we used more generic purposed metrics, namely Precision, Recall, and F1 score. Precision takes into account the number of attributes correctly classified to a given class compared to the number of characteristics correctly and incorrectly classified to that class, which is computed based on Equation (3). Precision measures the classifier’s correctness, and the relevance of positive classifications. Higher precision means a higher number of true positives and lower number of false positives.

(3) $$Precision=\frac{TP}{TP+FP}$$

To complement the understanding of Precision, some papers in the literature considers the Recall as shown in Equation (4). The recall is the true positive rate, i.e., the ratio of correctly predicted positive to the total number of actually positive observations.

(4) $$Recall=\frac{TP}{TP+FN}$$

F1-score is the weighted average of Precision and Recall as shown in Equation (5), where the first is the ratio of correctly predicted positive observations to the total predicted positive observations while the Second is the ratio of correctly predicted positive to the total number of actually positive observations.

(5) $$F1=2*\frac{precision*recall}{precision+recall}$$

### 4.2. Evaluation

**Figure 5:**
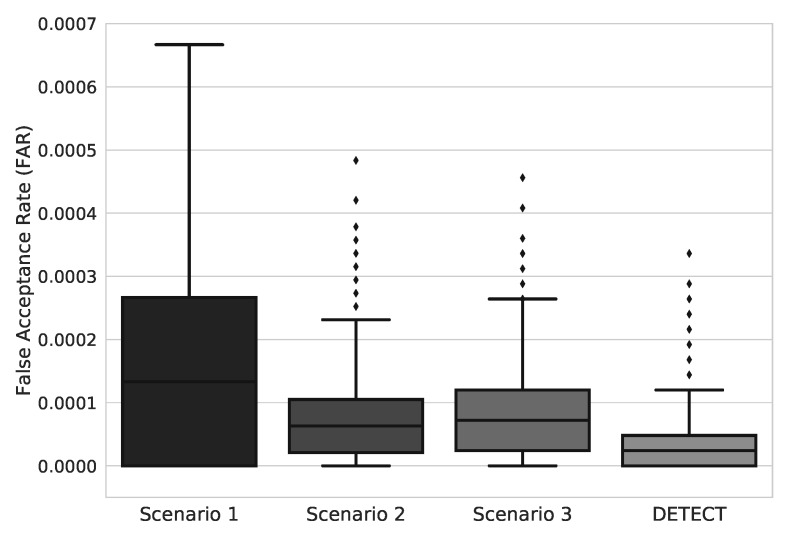
FAR results for each scenario of data improvement models.

For identification purposes, a few seconds of the collected ECG signal is passed to the classifier so it can decide which user the ECG signal would belong to (if any). In this sense, we performed a quantitative analysis to evaluate the FAR and FRR achieved for each scenario. Figure 5 shows the FAR results for the different user identification models considering the PHYSIONET challenge dataset with 1985 subjects. Subjects at scenario 1 had FAR values contained in the largest interquartile range and with highest values than any other scenario. As we increased data treatment the interquartile range narrowed, and FAR values became smaller (except for scenario 3 that had FAR values slightly higher than scenario 2). In the results from DETECT, we see the narrowest interquartile range and the smallest FAR values, which represent a low variance in FAR values and a low rate of incorrect identifications.

**Figure 6:**
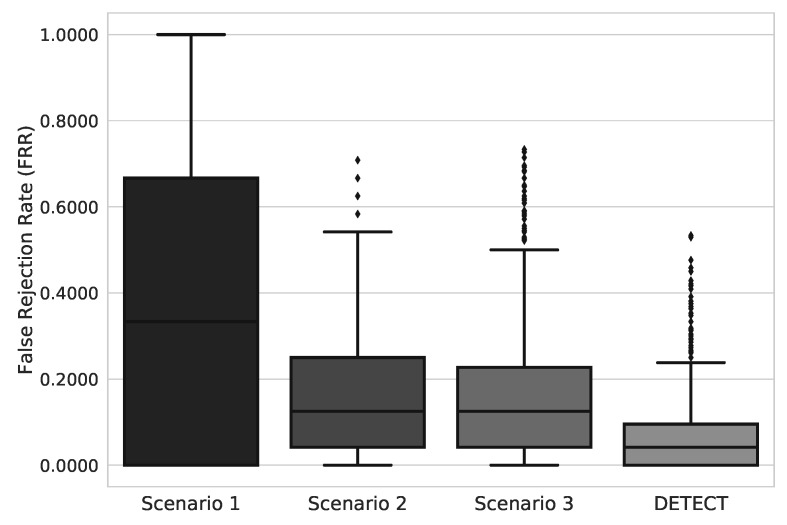
FRR results for each scenario of data improvement models.

Concerning FRR, as demonstrated in Figure 6, we can analyze a behavior much similar to what happened regarding false acceptance rates: Data treatment narrows the interquartile range and lower the rates. Subjects at scenario 1 had FRR values contained in the largest interquartile range and wit highest values than any other scenario. At DETECT, we see the narrowest interquartile range and the smallest FAR values, which represents a low variance in FRR values and a low rate of incorrect identifications. Concerned with the elevated number of subjects, we also evaluated the standard deviation of both FAR and FRR values for each step as seen in Table 3.

**Figure 7:**
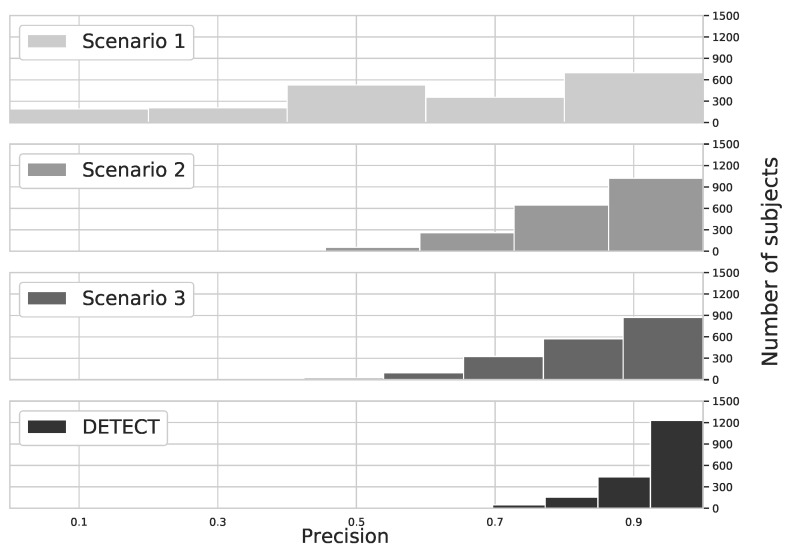
Precision distribution for the different user identification models.

In the continuous authentication scenario, the ECG would get as a stream and the metrics would be calculated considering successful attempts against all attempts during a certain time for each subject. In this sense, we performed a quantitative analysis to evaluate the precision achieved for each subject. Figure 7 shows the precision histogram for different user identification models based on ECG. We considered the PhysioNet Computing in Cardiology 2018 database with 1985 subjects. By analyzing the results, we can conclude that DETECT model provides a higher precision than other models, which means that the results are repeatable due to precision metrics consider true positive and false positive cases. Specifically, less than 25 subjects achieved a lower precision (i.e., precision 60% or less) considering DETECT model, which is less than 2% of the total population. On the other hand, more than 1200 subjects achieved precision higher than 90%, which is about 60% of the population. This is because our model considers only data with minimum quality, avoiding false positive caused by outliers.

The precision metric for data without any improvement (Scenario 1) presents a sparse result, where each of the 5 bins has a few hundred subjects each, which represents that the model has difficulty to precisely identify subjects due to a small number of examples. By increasing the number of examples with data augmentation (Scenario 2), and removing outliers (Scenario 3) we can see that bins associated with a high precision score are more filled and bins associated with a lower precision score are empty. At Scenario 2 more than 1200 subjects achieved at least a 70% precision score), whereas at Scenario 3 this number is even higher (close to 1400 subjects achieved at least a 70% precision score). And finally, using DETECT close to 1700 subjects wherein bins indicating at least a 70% precision score.

**Figure 8:**
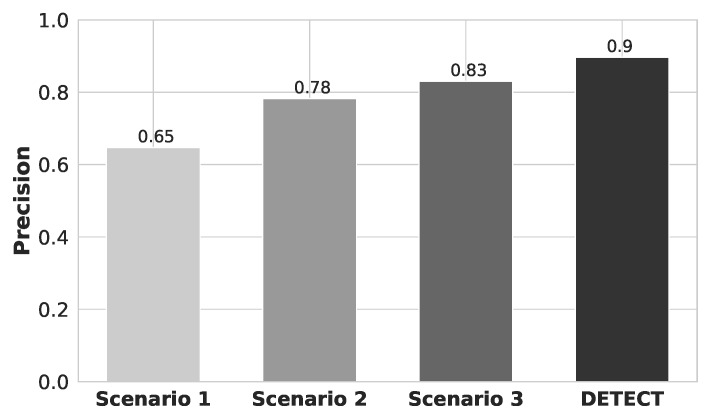
Precision results for each scenario of data improvement.

Figure 8 shows the precision results for each one of different data improvement scenarios. By analyzing the results, we conclude that DETECT achieved higher precision compared to the analyzed model. This is because a sub-segment is randomly extracted from the ECG stream, allowing us to increase the size of the data set. DETECT model also performed outlier removal and we increased the number of ECG features to evaluate the increasing of security metrics for user identification. Specifically, DETECT model increase the precision by 25%, 12%, and 7% compared to Scenario 1, Scenario 2, and Scenario 3, respectively. This means that DETECT model is able to provide a higher probability that a randomly selected retrieved user is relevant, i.e., how many of the returned hits were true positive, or how many of them were not found correctly, i.e., true negative.

**Figure 9:**
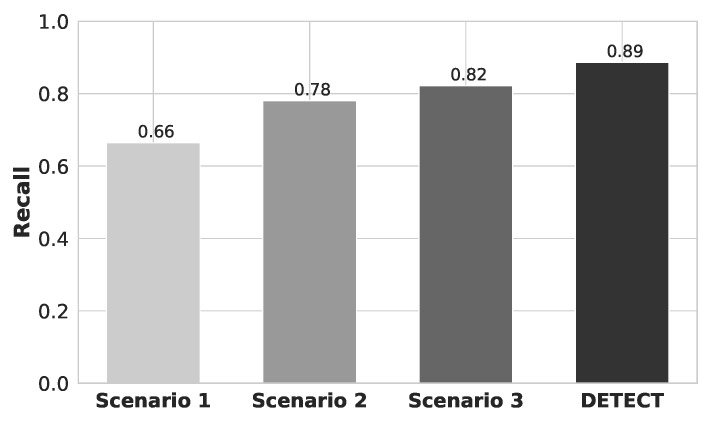
Recall results for each scenario of data improvement.

Figure 9 shows the recall results for each one of the different data improvement scenarios. By analyzing the results, we conclude that recall results follow almost the same ratio compared to Precision results. Each step of our proposed data improvement contributed to the overall increase of evaluated metrics. It is possible to notice that our data augmentation process was the step that contributed more. It makes sense if you consider that the data set size is one of the most important things to train a model [10]. The addition of new features was the second greater contributor. The increasing number of features can also increase the complexity of the model, which can result in a longer model fitting time. Specifically, in the first scenario (i.e., data without any improvement), we obtained an average recall of 66%. The second scenario (i.e., increasing the number of examples) achieved an average recall of 78%. The third scenario (i.e., increasing the number of examples and removing outliers) obtained an average recall of 82%. Finally, considering DETECT model (i.e., increasing the number of examples and removing outliers, and also considering features QRS Onset, Offset, and points P and S), we achieved an average recall of 89%. This means that DETECT has a higher probability that a randomly selected relevant user is retrieved in a search, i.e., how many of the true positives were recalled (found), or how many of the correct hits were also found.

**Figure 10:**
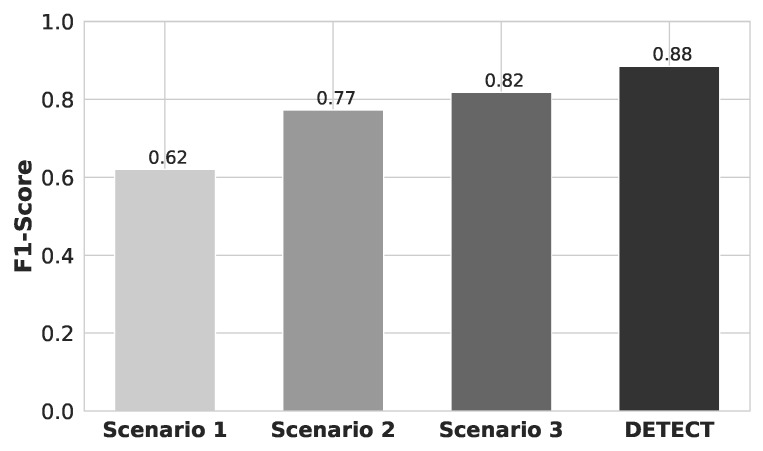
F1-score results for each scenario of data improvement.

Figure 10 shows the F1-score results for each one of different data improvement scenarios. Scenario 1, Scenario 2, Scenario 3, and the DETECT achieved a F1-score of 62.28%, 72.45%, 76.4%, 82.49%, respectively. Hence, DETECT has a higher ratio of correctly predicted positive to the total number of actually positive observations, i.e., indicates high values for both recall and precision.

**Figure 11:**
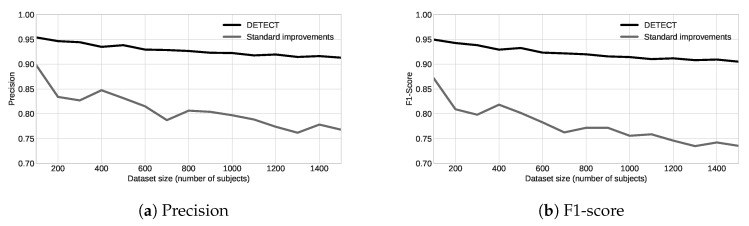
Results for different dataset size for the DETECT and standard model.

To evaluate how DETECT model would perform with a small data set, we divided it into subsets of hundreds and build a model with the same parameters as we did for the whole data set, as shown in Figure 11. By analyzing the results, we conclude that precision and F1-score reduce as soon as the number of target classes increases. For instance, precision starts around 78% with 100 subjects and ends close to 60% using 1500 for the scenario with an increased number of examples and outlier removal. This is because the ML model needs more features to generalize a great number of classes. On the other hand, DETECT model increased precision in 20% for 100 subjects, and 16% for 1500 subjects compared to results with fewer features. Similar behavior happens for the F1-score results.

## 5. Conclusions and Future Works

This article presented a data improvement model to elevate the baseline of FAR, FRR, precision, and f1 score of ECG-based identification systems. We used for the first time the dataset from PhysioNet Computing in Cardiology 2018 database. It allows assessing how the biometric system performs with around 1900 subjects. We observed that proposing a set of data improvement the metrics can improve considerably using the one-vs-rest classifier based on the random forest classifier. blueWithout any data improvement, mean false acceptance rates were 0.0194%, mean false rejection rates were 38.38% and approximately 600 subjects could be classified with at least 80% accuracy. After applying on data our proposed approach mean false acceptance rates were 83% smaller (0.0033%), mean false rejection rates were 83% smaller (6.40%) and approximately 1200 subjects could be classified with at least 80% accuracy. Even when working with a small number of subjects, a set of data improvement should be used, and our proposal achieve good results. For a subset of 100 subjects, we achieved 95% considering our model against 89% precision without our processing stages. We understand our set of methods could be applied to all dataset building process to improve the results. Our proposal is simple and easy to replicate in other databases and a direct comparison would not be fair, since most works use small databases as input. Thus, our comparison was in terms of how the results improved with each step of the data improvement.

As future works, an approach considering other signals and context information through services and other biomarkers would be a path to follow. A combination of passwords, face, ECG and new signals such as PPG should be investigated. We believe that multi-factor authentication using biometric signals is a trend to be explored soon.

## Figures and Tables

**Table 1:** Related Works Summary.

Paper	# of Subjects	# of Features	Acquisition Method or Database	Classifier	Metrics	Observation
Biel et al. [9]	20	10 to 360	Siemens Megacart	PCA built in SIMCA	100%	Fiducial Features and Feature Reduction
Camara et al. [13]	10	256	IMDs	DT, SVM, NN	93.5% 97.9%	Non-fiducial
Zhang and Wu [16]	85	9	PTB, MITDB, NSRDB	SVM, NN, LIBSVM	96.6% 97.7%	Used voting mechanism
Zhang, Y et al. [8]	100	10 + Feature space	MIT-BH	PCA + LDA	99%	Used hybrid approach
Zhang, Q et al. [11]	220	Wavelet Transform	CEBSDB, WECG, NSRDB, MITDB, VFDB, AFDB, STDB, FANTASIA	1D-CNN	96.5% 90.5% 93.5%	Used 1D-CNN
Labati et al. [18]	237	V vectors	E-HOL-03- 0202-003 and PTB	1D-CNN	100%acc EER = 3.81% EER = 3.37%	Preferred use EER and ROC
Zhang, Y et al. [19]	319	400	PTB, CEBSDB, NSRDB, MITDB	SVM, NNC	96.5–99.1% 95.4–97.8%	Used Inception and ResNet
Cao et al. [20]	N/A	8528 segments	Computing in Cardiology Challenge 2017	RNN, LSTM	F1 score: 0.82, 0.91, 0.84 and 0.70	Duplication, concatenation and resampling of ECG episodes
Pouryayevali et al. [22]	1012	feature space based on other works	UofTDB	LDA	Evaluate the increasing of population size	collected data in many positions stand sit tripod, and supine
Alotaiby et al. [21]	290	11 statistical features	PTB	RF	99.61%, 99.73% 99.76%, and 99.76%	used direct and band-based approaches.

**Table 2:** Features captured directly from ECG stream.

No.	Features
1	Mean Q Peak amplitude (μV)
2	Mean R Peak amplitude (μV)
3	Mean S Peak amplitude (μV)
4	Q Peak Standard Deviation
5	R Peak standard deviation
6	S Peak standard deviation
7	QRS amplitude (μV)
8	R wave duration (ms)
9	R-R Interval (ms)
10	Mean P Peak amplitude (μV)
11	Mean T Peak amplitude (μV)
12	Mean QS Distance
13	Mean QT Distance
14	Mean QRS onset amplitude (μV)
15	Mean QRS offset amplitude (μV)
16	Mean QT interval
17	Mean ST interval
18	Mean T wave
19	Mean PQ segment
20	Mean ST segment
21	Mean TP segment
22	Mean PP interval

**Table 3:** Mean FAR and FRR values and standard deviation in each data improvement models.

Scenario	FAR	Standard Deviation	FRR	Standard Deviation
Scenario 1	0.000194	0.000207	0.383813	0.312530
Scenario 2	0.000079	0.000069	0.156959	0.134858
Scenario 3	0.000080	0.000071	0.153801	0.143330
DETECT	0.000033	0.000042	0.064003	0.084876
